# Prevalence of Sexually Transmitted Infections and Human Immunodeficiency Virus in Transgender Persons: A Systematic Review

**DOI:** 10.1089/trgh.2019.0053

**Published:** 2020-06-08

**Authors:** Olivia T. Van Gerwen, Aditi Jani, Dustin M. Long, Erika L. Austin, Karen Musgrove, Christina A. Muzny

**Affiliations:** ^1^ Department of Medicine, Division of Infectious Diseases, University of Alabama at Birmingham, Birmingham, Alabama, USA.; ^2^ Department of Medicine, University of Alabama at Birmingham, Birmingham, Alabama, USA.; ^3^ Department of Biostatistics, School of Public Health, University of Alabama at Birmingham, Birmingham, Alabama, USA.; ^4^ Magic City Wellness Center, Birmingham AIDS Outreach, Birmingham, Alabama, USA.

**Keywords:** epidemiology, HIV/AIDS, sexually transmitted infections, transgender

## Abstract

**Purpose:** Despite reportedly high rates of human immunodeficiency virus (HIV) and other sexually transmitted infections (STIs) among transgender people, laboratory-proven prevalence of these infections in this population has not been systematically reviewed. We performed a systematic review and meta-analysis of the medical literature involving laboratory-proven HIV and STI diagnoses among transgender people.

**Methods:** A systematic review of the English literature regarding laboratory-proven HIV and/or STI testing in transgender populations within the last 50 years was performed. Preliminary meta-analyses assessing the prevalence of HIV and STIs among both transgender men and transgender women were performed. Given the heterogeneity of included studies, these analyses were difficult to interpret and not included in our results.

**Results:** Our literature review identified 25 studies, representing 11 countries. All of these studies included transgender women, with 9 (36%) including data on transgender men. HIV was the most commonly studied STI, with prevalence ranging from 0% to 49.6% in transgender women and 0% to 8.3% in transgender men. For syphilis, gonorrhea, and chlamydia, respectively, prevalence ranged from 1.4% to 50.4%, 2.1% to 19.1%, and 2.7% to 24.7% in transgender women and from 0% to 4.2%, 0% to 10.5%, and 1.2% to 11.1% in transgender men. Site-specific testing practices for gonorrhea and chlamydia were variable. No studies reported prevalence data on trichomoniasis.

**Conclusion:** The literature describing STIs and transgender people primarily focuses on transgender women and HIV. Data involving HIV and STIs among transgender men are lacking. These findings highlight opportunities for the future study of epidemiology of HIV/STIs in transgender men and the relevance of STIs in transgender people.

## Introduction

While it is estimated that 1.4 million adults in the United States identify as transgender, this population continues to be marginalized and underresearched.^1^ The visibility of the transgender community has increased significantly in recent years.^1,2^ The definition of “transgender” is constantly evolving, but typically refers to individuals whose gender identity does not match their sex assigned at birth.^3^ This enhanced visibility has highlighted the myriad health disparities affecting this community, including sexually transmitted infections (STIs) and human immunodeficiency virus (HIV).^4,5^

Particularly well studied is HIV in the trans feminine population.^6–10^ For transgender women, HIV rates are disproportionately high compared to other high-risk groups like men who have sex with men (MSM) and sexual partners of people living with HIV.^11^ This is hypothesized to be related to high-risk sexual behaviors such as commercial sex work, unprotected receptive anal intercourse, and multiple casual sex partners, which have been described as relatively common among transgender women.^6^ Unsafe needle injection practices associated with gender-affirming therapy (i.e., black market estrogen and silicone injections) have also been described as increasing the risk of HIV acquisition in transgender women.^12^ There is, however, a paucity of data for non-HIV STIs such as chlamydia, gonorrhea, syphilis, viral hepatitis, and herpes simplex virus (HSV), and how they affect transgender women.

In addition, little is known about the prevalence of any STIs, including HIV, in transgender men. In studies of transgender sexual health, inclusion of transgender men has been lacking as this population is historically difficult to recruit for research.^13,14^ Similar to transgender women, transgender men are emotionally and sexually attracted to people of all gender identities and can identify as straight, gay, bisexual, queer, or with another sexual orientation, resulting in a range of sexual risk behaviors among this group.^15,16^ One review found that a common goal of sexual behaviors among transgender men was affirmation of their post-transition masculinity, leading many to have receptive anal sex with cisgender men and avoidance of vaginal sex.^16^ Infrequent condom use is also described among transgender men; in one study that included 62 transgender men, 71% reported not using a condom during their last sexual encounter.^17^ Similarly, in a study of 23 transgender men in Boston, MA, 26% reported condomless or anonymous anal and vaginal sex.^18,19^ While cisgender MSM engage in condomless sex up to 57% of the time,^20^ sexual risk behaviors in transgender men still likely impact the STI milieu affecting this population and further study is needed.

Most research addressing the sexual health of transgender individuals focuses on sexual risk behaviors.^16^ Many of these studies utilize self-reported HIV and STI data as opposed to laboratory testing data.^19,21^ This makes it difficult to know the exact prevalence of these infections and, therefore, to formulate and implement HIV/STI prevention strategies for transgender people. We aimed to perform a systematic review and meta-analysis of the medical literature to characterize the epidemiology of HIV and STIs in both transgender men and women based on laboratory-confirmed testing data. Particularly, this analysis will help to elucidate the prevalence of bacterial, viral, and parasitic STIs in transgender men and women. There are a few examples of systematic reviews attempting to understand the prevalence of HIV/STIs in transgender populations,^22,23^ but none of these included only studies utilizing laboratory testing data. Given the aforementioned high-risk sexual behaviors described in transgender women, including participation in commercial sex work, we hypothesized that laboratory test-proven HIV and STI prevalence would be higher among transgender women compared to transgender men.

## Methods

### Search strategy

To compile a list of publications for possible inclusion in this systematic review of the literature, we searched the electronic databases of PubMed, Google Scholar, Embase, and Scopus for articles pertaining to HIV/STI prevalence in the transgender population using the following search terms: transgender, male-to-female, female-to-male, MTF, FTM, STI, STD, sexually transmitted infection, and sexually transmitted disease. In addition, the following combination query was used to identify potentially appropriate publications: ((transgender OR transsexual OR transvestite) AND (male-to-female OR female-to-male OR MTF OR FTM) AND (STI OR STD OR sexually transmitted infection OR sexually transmitted disease)). This search strategy was created in collaboration with an experienced health sciences librarian at our institution.

Given the myriad STIs we hoped to capture in our search, we chose to use the general terms of “STI,” “STD,” “sexually transmitted infection,” and “sexually transmitted disease,” as opposed to individual disease-specific terms to streamline our search strategy. This was possible due to the Medical Subject Headings (MeSH) database, which is integrated into PubMed. The MeSH thesaurus is a hierarchically organized vocabulary produced by the National Library of Medicine, which is used to index and catalog health-related literature.^24^ For example, when “sexually transmitted diseases” is searched, the MeSH functionality pulls articles that contain words associated with that search term, including a variety of specific diseases, including gonorrhea, chlamydia, syphilis, and herpes. In addition, when the search term “transgender” is used, literature containing the terms “transsexual” and “transvestite” are also captured.

Reference lists from selected publications were reviewed to identify articles for potential inclusion. IRB approval was not required for this study as it was a systematic review of the literature and no human subjects were enrolled. This review was not registered in a database of systematic reviews such as PROSPERO because data abstraction was completed before registration was pursued.

### Eligibility criteria

Searches were restricted to peer-reviewed, original research studies published in the English language within the past 50 years (between January 1968 and October 2018). To be included in this systematic review, studies had to include laboratory testing data for STIs, including HIV, gonorrhea, chlamydia, syphilis, viral hepatitis (Hepatitis A, B, and C), HSV-1 and HSV-2, or trichomonas, among transgender men and women. Testing for these infections had to be performed as part of a study protocol and reported. Studies were not excluded based on the method of testing as long as blood, urine, genital, or extragenital samples were collected and tested as specified by the study protocol. Studies including self-reported HIV/STI test results were excluded. If studies aggregated MSM together with transgender women, they were also excluded.

### Study selection

**FIG. 1.**
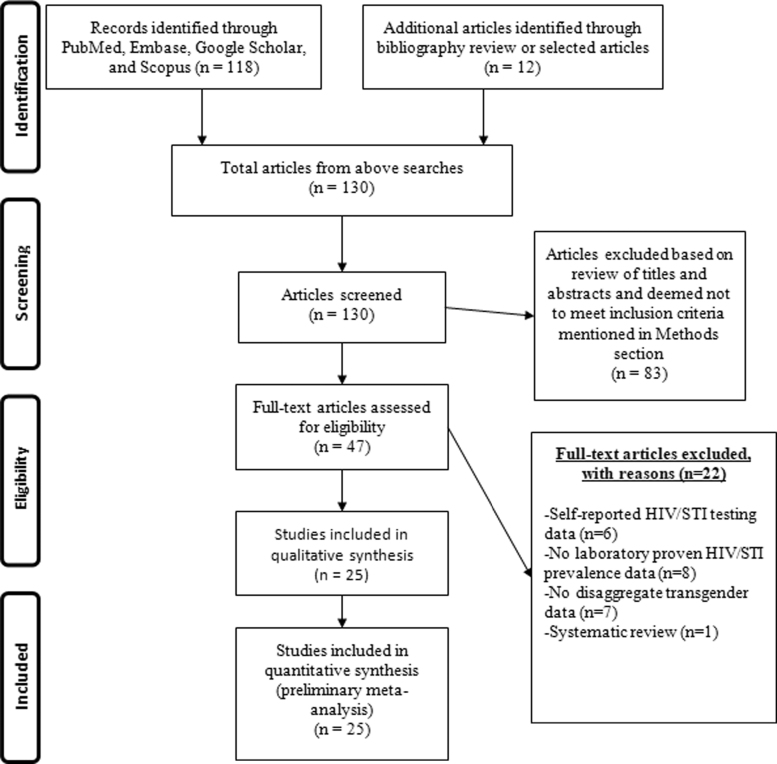
Flow diagram of study selection according to PRISMA Guidelines. PRISMA, preferred reporting items for systematic reviews and meta-analysis.

To determine article eligibility, authors O.T.V.G. and A.J. reviewed titles of articles yielded in the search to screen for relevance based on the aforementioned inclusion criteria. If deemed relevant, the abstract was scanned to further validate the meeting of inclusion criteria; if a determination could not be made from the abstract, full text review of the article was performed. All stages of the assessment of studies (identification, screening, assessment of eligibility, and inclusion) were performed by O.T.V.G. and A.J. If it was unclear whether or not an article was appropriate for inclusion, C.A.M. assisted in assessing articles for inclusion. In total, 130 abstracts were screened and 47 articles were selected for full text review. Of these, 22 were excluded because they did not meet our inclusion criteria, yielding 25 eligible articles. This process is summarized in Figure 1 through a preferred reporting items for systematic reviews and meta-analyses (PRISMA) flow diagram of article selection.^25^

### Data collection process

Before starting data collection, a Google Docs (Google, 2018) shared spreadsheet was created by author O.T.V.G. All co-authors were given access to this spreadsheet. The components of the spreadsheet were chosen based on agreement among authors and included laboratory testing on the following STIs: HIV, non-HIV viral STIs (viral hepatitis and HSV-1 and HSV-2), bacterial STIs (gonorrhea, chlamydia, and syphilis), and parasitic STIs (trichomonas). In addition, included articles were uploaded into a shared EndNote library (Clarviate Analytics, 2018).

### Analysis

Eligible articles were identified through the above search strategy between September 1, 2018, and November 1, 2018, by authors O.T.V.G. and A.J. Author C.A.M subsequently assisted in reviewing the articles found by the primary search. The following information was extracted from each article's text and input into a shared spreadsheet: (1) patient characteristics (including number, gender identity, participation in sex work, and sexual orientation), (2) laboratory testing methods for HIV and STIs, and (3) prevalence data of HIV and STIs.

**FIG. 2.**
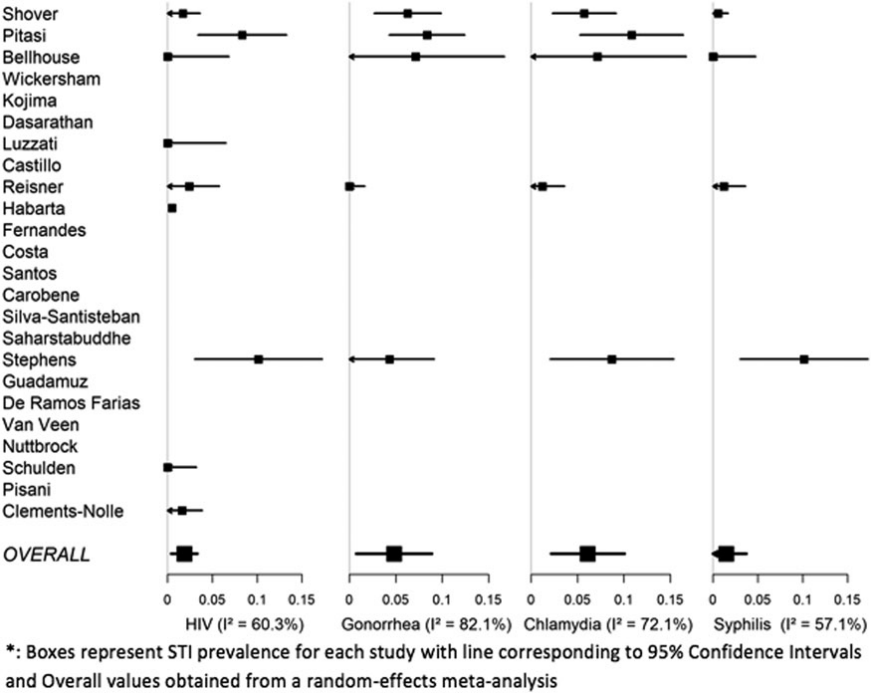
Forest plots of STI prevalence measures for transgender men. STI, sexually transmitted infection.

**FIG. 3.**
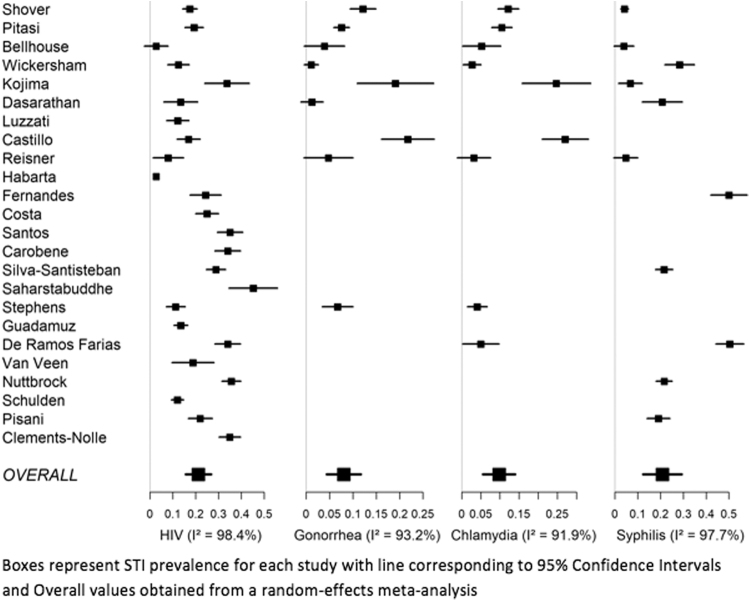
Forest plots of STI prevalence measures for transgender women.

To estimate the overall prevalence of HIV and each STI across studies, meta-analyses were performed using the meta package in R using random-effect models.^26^ HIV and each of the three most common STIs (syphilis, gonorrhea, and chlamydia) were considered separately for transgender men and transgender women. Once these analyses were performed, it was clear that the estimates were highly influenced by heterogeneity.^27,28^ *I*^2^ values for all analyses were >57% with all transgender women's STI meta-analyses having >90%, indicating that over 90% of the overall variability in STI prevalence estimation was due to between-study variability. Likewise, hypothesis tests for heterogeneity, Q tests, were significant for all models, except for syphilis in transgender men with *I*^2^=57% and *p*=0.07.^29^ This gives evidence that the variability between studies is due to other factors above and beyond simple random chance, which makes combining them into one statistic or analysis problematic. Therefore, we chose to exclude the full results for these meta-analyses and only present our systematic review of the literature on this topic. Figures 2 and 3 illustrate these results visually through forest plot and report *I*^2^ for each outcome. It is clear from these plots that the heterogeneity between studies is quite high for all outcomes, especially in transgender women. We also included the meta-analytic average for each outcome for reference, but are not confident in their validity.

## Results

This review includes 25 studies that met our inclusion criteria.^7,10,11,29–51^ Details regarding these studies and their characteristics can be seen in Table 1. The size of studies, including transgender women, varied from 63 to 13,154 people (median, 786) and those including transgender men ranged from 25 to 2364 people (median, 336). Studies from the included articles were conducted in 11 countries (United States, Australia, Malaysia, Peru, India, Italy, Brazil, Argentina, Thailand, the Netherlands, and Indonesia). There were nine studies conducted in the United States. All of the U.S. studies represented patient populations in urban settings, with data largely collected from academic medical centers in coastal cities. San Francisco, CA, was a primary study site in seven of the nine U.S. studies, with New York City, NY, and Boston, MA, also being represented in four and two studies, respectively.^11,30,31,37,38,41,45,49,50^ Only one multisite study presented data from the Southeastern United States, with a study site in Atlanta, GA.^31^ The non-U.S. studies included a variety of global regions, and while the majority revolved around urban areas, only one study from Argentina intentionally performed analyses on both urban and rural populations.^47^

**Table 1.** Description of 25 Studies of Laboratory Test-Proven HIV/Sexually Transmitted Infections in Transgender Persons

Source	Location	Study design	Study population	N	Transgender sample	HIV prevalence (%)	Non-HIV viral STI prevalence (%)	Bacterial STI prevalence (%)	Parasitic STI prevalence (%)
Shover et al.^30^	Los Angeles, CA	Cross-sectional analysis	CW, CM, TW, TM	19,933	TW = 551; TM = 175	TW 1%; TM 0%	HAV TW 0% TM 0% HBV TW 2% TM 0% HCV TW 4% TM 1%	GC TW 13% TM 7% CT TW 13% TM 7% Syphilis TW 21% TM 0%	n/a
Pitasi et al.^31^	Multiple sites, United States	Population-based survey	TW, TM, MSM, CW	626	TW = 506; TM = 120	TW 14.2%; TM 8.3%	n/a	GC TW 12.6% TM 10.5% CT TW 13.1% TM 7.7%	n/a
Bellhouse et al.^32^	Melbourne, Australia	Retrospective cohort study	TW, TM	133	TW = 77; TM = 28	TW 9%; TM 4%	n/a	GC TW 4% TM 9% CT TW 6% TM 8% Syphilis TW 8% TM 0%	n/a
Wickersham et al.^7^	Kuala Lumpur, Malaysia	Cross-sectional analysis	CW, TW	492	TW = 193	TW 12.4%	n/a	GC 1.1% CT 2.7% Syphilis 28.3%	n/a
Kojima et al.^33^	Lima, Peru	Observational cohort study	TW, MSM	401	TW = 89	TW 33.7%	n/a	Any GC 19.1% Rectal GC 11.2% Pharyngeal GC 7.9% Any CT 24.7% Rectal CT 13.5% Pharyngeal CT 11.2% Syphilis 6.7%	n/a
Dasarathan and Kalaivani^10^	Chennai, India	Retrospective study	TW	82	TW = 82	13.4%	n/a	Syphilis 20.7%	n/a
Luzzati et al.^34^	Trieste, Italy	Retrospective observational study	TW, TM	243	TW = 218; TM = 25	TW 12.1%; TM 0%	HBV TW 4.6% TM 4% HCV TW 3.7% TM 8%	n/a	n/a
Leon et al.^35^	Lima, Peru	Cross-sectional analysis	TW, MSM	718	TW = 208	n/a	n/a	Rectal GC 12.3% Pharyngeal GC 9.6% Rectal CT 20.1% Pharyngeal CT 6.7%	n/a
Castillo et al.^36^	Lima, Peru	Prospective cohort study	TW, MSM	718	TW = 207	16.9%	HSV 80.7%	Rectal GC 12.3% Pharyngeal GC 9.7% Rectal CT 20.2% Pharyngeal CT 6.8%	n/a
Reisner et al.^37^	Boston, MA	Retrospective observational study	TW, TM	145	TW = 63; TM = 82	TW 7.9%; TM 2.4%	HSV TW 2.1% TM 1.2% HCV TW 3.2% TM 2.4%	GC TW 2.1% TM 0% CT TW 3.2% TM 1.2% Syphilis TW 4.8% TM 1.2%	n/a
Habarta et al.^38^	Multisite, United States	Cross-sectional analysis	TW, TM, CM, CSW	15,518	TW = 13,154; TM = 2364	TW 2.7%; TM 0.9%	n/a	n/a	n/a
Fernandes et al.^39^	Campo Grande, Central Brazil	Cross-sectional analysis	TW, MSM	430	TW = 152	24.4%	n/a	Syphilis 50%	n/a
Costa et al.^40^	Southern Brazil	Cross-sectional analysis	TW	284	TW = 284	25%	n/a	n/a	n/a
Santos et al.^41^	San Francisco, CA	Cross-sectional analysis	TW	314	TW = 314	35%	n/a	n/a	n/a
Carobene et al.^42^	Argentina	Cross-sectional analysis	TW	273	TW = 273	34%	HBV 40.2%; HCV 4.5%	n/a	n/a
Silva-Santisteban et al.^43^	Lima, Peru	Cross-sectional analysis	TW	450	TW = 450	29.6%	HSV 79.4%	Syphilis 22.9%	n/a
Sahastrabuddhe et al.^44^	Pune, India	Cross-sectional analysis	TW, MSM, CM	14,100	TW = 84	45%	n/a	Syphilis 10.3%	n/a
Stephens et al.^45^	San Francisco, CA	Cross-sectional analysis	TW, TM	292	TW = 223; TM = 69	TW 1.4%; TM 2.9%	n/a	Rectal GC TW 6.3% TM 3.7% Pharyngeal GC TW 3.5% TM 4.9% Urogenital GC TW 2% TM 0% Rectal CT TW 4.2% TM 11.1% Pharyngeal CT TW 2.1% TM 4.9% Urogenital CT TW 0% TM 4.2% Syphilis TW 4.2% TM 4.2%	n/a
Guadamuz et al.^46^	Multisite, Thailand	Cross-sectional analysis	TW	474	TW = 474	13.5%	n/a	n/a	n/a
Dos Ramos Farías et al.^47^	Multisite, Argentina	Cross-sectional analysis	TW	387	TW = 273	34.1%	HBV 40.2%; HCV 4.5%	Rectal CT 5% Syphilis 50.4%	n/a
Van Veen et al.^48^	Multisite, Netherlands	Cross-sectional analysis	TW, CSW	557	TW = 70	18.8%	n/a	n/a	n/a
Nuttbrock et al.^49^	New York, NY	Cross-sectional/longitudinal study	TW	517	TW = 517	3.5%; 49.6%; 48.1%^a^	HBV 6.5%; 36%; 35.5%^a^; HCV 3.6%; 15.7%; 7.4%^a^	Syphilis 1.4%; 21.6%; 14.7%^a^	n/a
Schulden et al.^50^	Multisite, United States	Cross-sectional analysis	TW, TM	601	TW = 559; TM = 42	TW 12%; TM 0%	n/a	n/a	n/a
Pisani et al.^51^	Jakarta, Indonesia	Cross-sectional analysis	TW, MSM	770	TW = 241	22%	n/a	Syphilis 28.2%	n/a
Clements-Nolle et al.^11^	San Francisco, CA	Cross-sectional analysis	TW, TM	515	TW = 392; TM = 123	TW 35%; TM 2%	n/a	n/a	n/a

CM, cisgender men; CSW, commercial sex workers; CT, chlamydia; CW, cisgender women; GC, gonorrhea; HAV, hepatitis A virus; HBV, hepatitis B virus; HCV, hepatitis C virus; HIV, human immunodeficiency virus; HSV, herpes simplex virus; MSM, men who have sex with men; STI, sexually transmitted infection; TM, transgender men; TW, transgender women.

^a^ Stratified by race, with prevalence reported for Caucasians; Hispanics; and African-Americans, respectively.

All 25 studies reported data on transgender women, with only 9 (36%) reporting data on transgender men. Of the studies investigating STIs in transgender women, four were focused on commercial sex workers.^7,42,47,48^ In fact, of the studies investigating transgender women, 4 (16%) exclusively evaluated commercial sex workers. Research involving transgender populations has moved away from grouping commercial sex workers and transgender women into the same study population, with most of these studies being conducted in 2013 or earlier.^42,47,48^

The most studied STI was HIV, with 24 (96%) of the eligible studies citing HIV prevalence data. Of these, 7 (28%) exclusively evaluated HIV and did not comment on study results for any other STI.^11,38,40,41,46,50^ In transgender women, the prevalence of HIV across all studies testing ranged from 0% to 49.6%, while in transgender men, the range was 0% to 8.3%. Of non-HIV STIs, syphilis was the most studied infection, with 13 (52%) studies reporting data. The prevalence range for syphilis in transgender women was 1.4% to 50.4% and in transgender men was 0% to 4.2%. No studies reported data for trichomoniasis in transgender people.

Regarding gonorrhea and chlamydia, 10 (40%) studies presented testing data. Of those, only five reported both urogenital and extragenital (e.g., pharyngeal and rectal) results. Even in these studies, screening practices were not uniform, with some studies failing to delineate which sites they were testing. In five of the studies where gonorrhea or chlamydia testing was reported, any positive test of any site qualified as diagnostic of that infection, but sites were not specified in their reporting.^7,30–32,37^ The overall prevalence of gonorrhea ranged from 2.1% to 19.1% in transgender women and 0% to 10.5% in transgender men. For chlamydia, prevalence ranged from 2.7% to 24.7% in transgender women and from 1.2% to 11.1% in transgender men.

Data on viral non-HIV STIs were reported in eight studies. Viral hepatitis data were included in six of these studies, with one reporting on hepatitis A virus (HAV), five reporting on hepatitis B virus (HBV), and six reporting on hepatitis C virus (HCV).^30,34,37,42,47,49^ In the one study that included HAV, the prevalence was found to be 0% among a transgender cohort of 726 participants, which included both transgender men and women.^30^ The prevalence of HBV ranged from 2% to 40.2% in transgender women and from 0% to 4% in transgender men. For HCV, the prevalence ranged from 3.2% to 15.7% in transgender women and from 1% to 8% in transgender men. HSV data were reported in 3 studies, all of which used serological testing for HSV; the prevalence in transgender women ranged from 2.1% to 80.7%.^36,37,43^ Only one study included HSV data on transgender men, which noted a prevalence of 1.2%.^37^ Two of the studies tested for HSV-2 only^36,43,52^ and the third study, which is the only one that mentioned transgender men, did not provide typing information about HSV serologies.^37^

## Discussion

Our systematic review revealed that the current literature involving laboratory proven HIV and STIs in transgender people has primarily been conducted in transgender women. Largely, the trans feminine community has been studied in the context of study participants also being commercial sex workers. While this is an important and highly vulnerable population, their HIV and STI data are not generalizable to all transgender women. Traditionally, one of the more visible subsets of the trans feminine population has been those involved in commercial sex work, but in the National Transgender Discrimination Survey (NTDS) of 2008–2009, 13.1% of transgender men indicated lifetime participation in commercial sex work.^53^ Along similar lines, trans masculine individuals have been grossly understudied; thus, very little is known about the prevalence of STIs in this population. There are multiple explanations for this underrepresentation. First, existing evidence suggests that the prevalence of HIV and STIs overall in transgender men is lower compared with transgender women.^30,31,37,54^ Many transgender men as well as health care providers caring for them also perceive that they are not at high risk of contracting HIV or STIs, which may in turn affect HIV/STI testing rates.^55^

The study sites of included articles, particularly those in the United States, disproportionately represented urban-dwelling transgender individuals. Little is known about transgender people who live in rural or remote areas, and based on this review, little is known regarding their HIV and STI prevalence. The current U.S. studies of STI prevalence are also regionally biased, favoring coastal cities like San Francisco, CA, Boston, MA, and New York City, NY. Aside from one multicenter study, including a study site in Atlanta, GA, which was found in our search, there is scant HIV and STI testing and prevalence data in the Southeastern United States for transgender individuals.^31^According to 2018 surveillance data from the Centers for Disease Control and Prevention (CDC), incident cases of gonorrhea, chlamydia, syphilis, and HIV were all highest in the Southeastern United States compared to all other regions.^53,56^ Given the significant impact of HIV and STI in the Southeastern United States, the lack of testing data for transgender individuals in the region as well as in rural areas merits further study to better understand the epidemiology of these diseases regionally.

HIV is by far the best studied infection of all those included in this review, with 96% of articles reporting testing data. Our review found HIV prevalence to be up to 49.6% in transgender women, which is consistent with the reportedly high HIV burden in this population.^57,58^ As mentioned above, transgender women were disproportionately better studied than transgender men, who were found to have up to 8.3% prevalence of HIV. This high burden of HIV among transgender women is alarming, but it is important to keep in mind that these data were highly variable across a small number of studies. Despite both CDC and the U.S. Preventive Services Task Force (USPSTF) recommendations for universal HIV screening in all adults and at least annual screening in high-risk adults, testing among transgender men and women is low.^59,60^ As per the CDC, only 35.6% of transgender women and 31.6% of transgender men have ever had an HIV test in their lifetime.^61^ Historically, risk of HIV acquisition in transgender men has been downplayed. However, multiple HIV risk factors have been associated with transgender men, including lack of access to intramuscular needles for testosterone injections, unprotected sex, and belief within the community that they are not at risk of infection.^62^ In addition, some qualitative studies utilizing self-reported HIV status have reported that transgender MSM may have a higher HIV prevalence than cisgender males and females.^18,19^ More research is needed to explore how transgender men are affected by HIV, particularly by way of behavioral analyses that could help guide preventive interventions.

Trichomoniasis is the most common non-viral STI worldwide.^63^ None of the studies included in our review contained testing data for trichomoniasis, but it is important to understand how this STI affects the transgender population, given the potential sequelae of untreated infection, such as serious reproductive morbidity (i.e., vaginitis, cervicitis, urethritis, and pelvic inflammatory disease), poor birth outcomes (i.e., premature rupture of membranes, low birth weight, and preterm delivery), and amplified HIV transmission.^64–66^ Current guidelines from the CDC recommend consideration of screening for trichomoniasis in patients in high-prevalence settings or for asymptomatic persons at high risk for infection (e.g., persons with multiple sex partners, exchanging money for sex, illicit drug use, or a history of STI). This could certainly apply to both transgender men and transgender women, but the data are lacking about whether or not screening^59^ these high-risk individuals has an impact on reduction of adverse health outcomes.^67^ More study is needed in this area and transgender individuals should be included in future studies informing screening guidelines.

Gonorrhea and chlamydia testing data, while mentioned in almost half of the studies included, were often difficult to interpret. Given the predilection of these infections to affect any mucosal site they come into contact with during sexual activity, it is important to consider screening and diagnostic testing at whichever sites the patient reports sexual contact.^68–71^ As mentioned above, more than half of the studies reporting on gonorrhea and chlamydia did not perform testing beyond the urogenital tract and of the others that did, they practiced inconsistent sampling practices and reporting of results. Currently, multisite testing and screening for gonorrhea and chlamydia are not routine for populations other than MSM.^59^ However, transgender men and transgender women often participate in sexual behaviors such as anal receptive intercourse and oral intercourse as do cisgender MSM, cisgender women who have sex with women, and cisgender men who have sex with women.^16,72^ Therefore, they may also be at risk of extragenital infection of these bacterial STIs and more research is needed to determine optimal screening and testing practices for these populations.

Of the non-HIV viral STIs included in this review, HCV was the most commonly studied. HCV was more prevalent in transgender women than transgender men. Sexual transmission of HCV through behaviors such as fisting (i.e., brachioanal sex) and receptive anal intercourse has been described in MSM populations,^73,74^ but the virus is generally more commonly acquired by nonsexual means of transmission like intravenous drug use.^75^ More study is needed to better understand how sexual practices might influence HCV acquisition in transgender individuals. HAV and HBV are more likely to be transmitted sexually than HCV, particularly when sexual behaviors involve fecal oral contamination (i.e., participation in both anal and oral sex).^75,76^ This could explain why transgender women may have a higher prevalence of HBV if they are engaging in high-risk receptive anal sex (i.e., in the setting of commercial sex work).^75^ The small number of studies citing these infections, however, makes it difficult to draw conclusions from the existing literature, so additional study of how viral hepatitis affects transgender individuals is needed.

Regarding HSV, two of the three studies collected HSV-2 serologies, which is typically associated with sexual transmission.^36,43,52^ None of these studies reported HSV-1 data. One study did not report serotype, so it is difficult to interpret the significance of reported HSV findings from this study.^37^ The seroprevalence of HSV-2 noted in this review ranged from 37.5% and 80.7%, which is higher than the seroprevalence of the general population (reported to be 12.1% in 2015–2016).^56^ As an STI, genital HSV infection is typically a clinical diagnosis and is not a nationally notifiable condition, so most people with genital HSV infection have not received a diagnosis.^77^ Serologies of HSV-2 are not useful in clinical practice and are mainly used in epidemiological studies.^78^ It is difficult to use the serological data from these few studies to draw conclusions about the impact of genital HSV infection on the transgender population, but higher seroprevalence could suggest increased exposure in this community.

We encountered several limitations when conducting this study. First, defining the search terms for this review was difficult for multiple reasons. Defining “transgender” with search terms is inherently difficult, given the community's heterogeneity and the evolution of how the community has defined itself over the last few decades.^3^ As noted in the Methods section, however, we believe that the combination search query used was able to adequately capture both historical and contemporary terminology regarding gender identity as well as a variety of specific STIs. In addition, while MeSH terms enabled us to capture the majority of common STIs with our search strategy, it is possible that literature describing less common STIs was missed, especially since MeSH terms are not necessarily retroactive. An alternative strategy that may have resulted in a more robust search would have been to include search terms for individual STIs; this should be included in future research efforts. Second, this review aimed to characterize HIV/STI prevalence through laboratory testing in transgender people, but search terms used, as well as the inclusion criteria of most studies included, did not capture gender nonconforming members of the lesbian, gay, bisexual, transgender, queer (LGBTQ) community such as gender fluid or genderqueer individuals. These individuals likely share a similarly diverse landscape of sexual practices and orientations to the transgender community, and are therefore important to include in future sexual health studies aimed at understanding STIs in underrepresented sexual minorities.^79^ These challenges highlight the need for researchers in the STI field to better tailor their method of collecting patient demographics regarding their gender identity. Third, we recognize that only including laboratory testing data for HIV and STIs in transgender individuals may not fully capture the full burden experienced by this population, given the aforementioned low rates of testing in these populations.

Another major limitation involves the heterogeneity of testing performed in the included studies. For example, in the four studies that included HSV data, one did not include any typed HSV data, making it difficult to draw conclusions about the prevalence of type-specific infection. Likewise, it was very difficult to draw conclusions based on the extragenital bacterial STI data as each study collected and reported their results differently. This lack of methodological uniformity between studies made it impossible to perform a useful meta-analysis with our findings. Human papilloma virus (HPV) is a common viral STI and was not included in this systematic review. The diagnosis is HPV is typically made clinically by the presence of anogenital warts, and therefore, does not meet the inclusion criteria for testing for this study. HPV can also be detected by Papanicolaou (Pap) testing, which should be performed routinely in transgender men who have not undergone surgery to remove their uteri and cervices. HPV testing can also be performed by anal Pap testing in individuals at high risk of anal infection, including transgender women who engage in receptive anal sex.^80^ Our search strategy for this review did not reveal any studies including such data, so a dedicated systematic review with an individualized search strategy is needed outside of this study.

## Conclusion

Currently, the literature involving STIs in transgender people is primarily focused on the trans feminine community and testing for HIV. While there are some data for bacterial STIs, testing patterns were variable, especially for gonorrhea and chlamydia. From the available data, STIs appear to be more prevalent in transgender women than their trans masculine counterparts. This finding, however, is not likely representative of the trans feminine community as a large proportion of studies only included commercial sex workers. There is a marked paucity in studies involving transgender men; therefore, it is difficult to know how truly representative these data are. These gaps in the literature present numerous opportunities for future studies, particularly involving the epidemiology of STIs in the Southeastern United States and transgender men, as well as the relevance of extragenital bacterial, parasitic, and non-HIV viral STIs in all transgender people.

## Abbreviations Used

CDC: Centers for Disease Control and Prevention
FTM: female-to-male
HAV: hepatitis A virus
HBV: hepatitis B virus
HCV: hepatitis C virus
HIV: human immunodeficiency virus
HPV: human papilloma virus
HSV: herpes simplex virus
MeSH: Medical Subject Headings
MSM: men who have sex with men
MTF: male-to-female
Pap: Papanicolaou
STD: sexually transmitted disease
STI: sexually transmitted infection
TV: trichomoniasis
